# Lessons of the COVID-19 Pandemic for Ambulance Service in Kazakhstan

**DOI:** 10.3390/healthcare12161568

**Published:** 2024-08-08

**Authors:** Assylzhan Messova, Lyudmila Pivina, Diana Ygiyeva, Gulnara Batenova, Almas Dyussupov, Ulzhan Jamedinova, Marat Syzdykbayev, Saltanat Adilgozhina, Arman Bayanbaev

**Affiliations:** 1 Department of Emergency Medicine, Semey Medical University, Semey 071400, Kazakhstan; diana.ygiyeva@smu.edu.kz (D.Y.); gulnara.batenova@smu.edu.kz (G.B.); almas.dyussupov@smu.edu.kz (A.D.); 2 Department of Epidemiology and Biostatistics, Semey Medical University, Semey 071400, Kazakhstan; ulzhan.jamedinova@smu.edu.kz; 3 Department of Anesthesiology and Reanimatology, Semey Medical University, Semey 071400, Kazakhstan; marat.syzdykbayev@smu.edu.kz; 4 Department of Family Medicine, Semey Medical University, Semey 071400, Kazakhstan; saltanat.adilgozhina@smu.edu.kz; 5 National Coordinating Center for Emergency Assistance, Astana 10000, Kazakhstan; astana077@gmail.com

**Keywords:** COVID-19, emergency, ambulance service, Kazakhstan, consultation

## Abstract

Background: Emergency medical services (EMS) are intended to provide people with immediate, effective, and safe access to the healthcare system. The effects of pandemics on emergency medical services (EMS) have not been studied sufficiently. The aim of this paper is to assess the frequency and structure of calls at an ambulance station in Kazakhstan during the period of 2019–2023. Methods: A retrospective analysis was conducted to estimate the incidence of emergency assistance cases from 2019 to 2023. Results: An analysis of the structure and number of ambulance calls before the pandemic, during the pandemic, and post-pandemic period did not reveal significant changes, except for calls in urgency category IV. Patients of urgency category IV handled by an ambulance decreased by 2 and 1.7 times in 2020 and 2021, respectively, which appears to be related to quarantine measures. In 2022 and 2023, category IV calls were 4.7 and 4.5 times higher than in 2019. Conclusions: This study’s findings suggest no changes in the dynamics of ambulance calls, except urgency category IV calls. The number of category IV urgent calls decreased significantly during the COVID-19 pandemic and increased in the post-pandemic period.

## 1. Introduction

Emergency medical services (EMS) are important component of a national healthcare system [1]. During pandemics, the national EMS must balance the ongoing demands of regular emergency cases with the additional needs brought on by the crises. In Kazakhstan, this role is performed by the National Coordinating Center of Emergency Medicine (NCCEM). The main role of the NCCEM is to improve the quality and availability of emergency medical care to reduce mortality and disability in the Republic of Kazakhstan. The NCCEM operates a fleet of about 1500 ambulances spread throughout the country. In addition to the ambulances, more than 30 units of air transport are used; medical aviation carried out from three to fifteen flights per day [2].

Kazakhstan has a small population (19 million residents), and remains one of the most sparsely populated countries. The republic ranks ninth in terms of territory, but in terms of population, according to the analysis of the UN Population Fund, there were only 6.8 people per square kilometer in 2019 [3]. In this regard, ambulances often serve large areas and the assistance of air ambulances is required. The largest share of flights is carried out to provide medical care in cases of injuries, including those caused by road accidents, as well as pathologies of childhood, diseases of the circulatory system, pathologies of newborns, obstetric and gynecological pathologies, and surgical, therapeutic, and other diseases. Mobile teams of republican or regional levels carry out the provision of medical care. The full-time mobile medical team includes a doctor and a paramedic or only paramedics; it is formed by the medical profile of the patients. If necessary, by significance, qualified specialists from large organizations, cities of republican significance and the capital, as well as educational and scientific organizations can be involved.

The COVID-19 pandemic has become the biggest health challenge worldwide. The load on almost all segments of the medical system, especially on first aid services, increased significantly. It has required a quick revision of the usual work algorithms. Prior reports on the number of EMS calls during a pandemic compared to post-pandemic were extremely few [4,5,6]. 

### Response to COVID-19 in Kazakhstan 

The first COVID-19 case in Kazakhstan was diagnosed on 13 March 2020 [7]. To prevent the spread of the disease, a state of emergency was introduced from 16 March to 11 May 2020. Restrictions were implemented on entry and exit from the country; quarantine or other restrictive measures were introduced in all regions, and the activities of large non-food retail outlets, cinemas, and other places with large crowds of people were stopped [8]. On 5 July 2020, a strict isolation regime began to operate in Kazakhstan. All facilities were closed, except for grocery stores, pharmacies, cafes (while maintaining social distancing), and airports (for domestic flights). Then, from 17 August 2020, Kazakhstan approved a plan for the phased lifting of quarantine. To provide timely medical care to patients with coronavirus infection, as of 17 March 2020, 248 infectious diseases hospitals were involved in the Republic of Kazakhstan, with a bed capacity of 23,462 beds [9].

According to the Johns Hopkins University data, four waves of coronavirus infection were observed in Kazakhstan; the first wave had a peak in July 2020, when 18,757 new cases of coronavirus infection were detected per day, the second wave in the winter of 2020–2021 (an average of 1300 cases of positive polymerase chain reaction (PCR) tests per day was detected), and the third wave in March 2021 with the number of new cases reaching 1500–3000 per day [10]. The peak of the fourth wave of coronavirus infection was observed in August 2021, associated with the spread of the Delta variant of the coronavirus. During the last increase in incidence at its peak, up to 7800 cases were recorded per day [11].

The COVID-19 call centers were opened, and citizens of the Republic of Kazakhstan could call 24 h a day to find out all the information about current situations on coronavirus incidence, as well as preventive measures and the main symptoms of diseases. They were created as a part of the regional/city emergency medical stations, which had operational communication with the service of the internal affairs and civil protection of the population in the regions, EMS substations, EMS departments at Primary Health Care Institutions, and emergency departments of medical organizations. In the call centers, consultations with the population were carried out by consulting doctors, senior doctors of regional/city emergency medical services, experienced paramedics and paramedical workers, and residents. Call center specialists informed the population on the provision of medical care at the pre-hospital stage and answered frequently asked questions related to the coronavirus infection. In the presence of such symptoms as headache, runny nose, chest pain, fever, tachycardia and rapid breathing, calls were redirected to the dispatcher 103.

The most effective method of combating coronavirus infection is vaccination. The mass vaccination campaign against COVID-19 began in Kazakhstan on 1 February 2021. In Kazakhstan, the local vaccine QazVac (Kazakhstan), as well as Sputnik V (Russia), Sinopharm (China), Sinovac (China), and Pfizer (America) were used. Currently, 55.35% of the population has received full vaccination [11]. There is a lack of research on the functioning of the emergency medical care system in Kazakhstan during the pandemic, which is necessary to learn lessons for the future.

This study aims to characterize the work of emergency medical services during the coronavirus pandemic and make recommendations to be applied for future preparedness.

## 2. Materials and Methods

### 2.1. Study Design

This study is a descriptive, population-based cross-sectional study of EMS work in the Republic of Kazakhstan before, during, and after the pandemic. Information on emergency medical calls was provided by the ambulance stations of 17 regions and three cities of republican significance of the Republic Kazakhstan. 

#### Ambulance Service in Kazakhstan

In the Republic of Kazakhstan, the ambulance service is structured as a centralized system. The structure of the ambulance service in Kazakhstan consists of a three-tier system, including ambulance stations, ambulance teams, and dispatch centers, provided by the government. The Republican Emergency Medical Center (RCC) of the Ministry of Healthcare of Kazakhstan is responsible for managing, planning, and coordinating the work of ambulance services throughout the country. Regional ambulance stations provide emergency medical care to their regions’ populations [12]. 

The emergency medical station dispatcher receives calls from citizens at the “103” remote control in case of health problems; the call processing time from the moment it is received by the emergency medical service dispatcher is five minutes, during which the call is sorted by category of urgency. There is also a call hold system by the dispatcher, which allows them to provide to assist until the ambulance arrives. The dispatcher explains over the phone how to provide emergency assistance. The dispatcher of the ambulance station performed the following actions: receiving all calls on the remote control 103; triage (screening) by call urgency category; using an automated system, transfering the calls of first, second, and third categories of urgency to the ambulance teams. The calls of the fourth category of urgency were transferred to the paramedical and medical teams of the ambulance and primary health care organizations. According to the Rules for the provision of emergency medical care, approved by Order of the Minister of Health of the Republic of Kazakhstan (3 July 2017 No. 450), all calls are divided into 4 (four) categories of urgency [13]. 

The first level of urgency signifies an immediate threat to life requiring emergency medical attention.

The second group relates to a potential threat to life in the absence of medical intervention, whereas the third category refers to a potential harm to health. The fourth category refers to a patient’s state caused by an acute sickness or worsening of a chronic disease that does not involve sudden and pronounced organ and system violations and does not provide an immediate or possible threat to the patient’s life.

The time of arrival of the EMS team from the moment of receiving a call should be up to 10 min for the first category of urgency, up to 15 min for the second category, up to 30 min for the third category, and less than 60 min for the fourth category of urgency (in such cases departure was carried out at the outpatient clinic). Upon receipt of a call of the fourth category of urgency, the ambulance dispatcher calls the brigade of the ambulance department at PHC using an automatic control system. In the case of a call, when there is no need for an emergency response team to attend a call at home, the dispatcher forwards the call to the call center.

The ambulance team, using the clinical protocol for treatment and diagnosis, determines the route of the patient’s movement depending on his condition according to the algorithm. In the case of a patient who does not need hospitalization, the ambulance team or the ambulance department at PHC provides medical recommendations for the provision of medical care in the PHC organization (at the place of residence). Before transporting the patient to the hospital, the ambulance team checks with the dispatch service about the availability of vacant places in the corresponding hospital and notifies the hospital emergency department of the upcoming hospitalization.

The number of ambulance stations in Kazakhstan has increased significantly over the past 10 years; at the moment, there are over 400 stations and substations in total [12]. In our study we included all ambulance usage, even if an ambulance was used to transfer a patient from one hospital to another.

### 2.2. Ethical Approval Details

The study has approval of the Local Ethics Commission of the Semey Medical University on 16 March 2022. Information on emergency medical calls was provided by the National Center for Emergency Medicine. All ambulance stations within 17 regions and 3 cities of republican significance provide monthly and annual reports with the structure of calls to the National Center for Emergency Medicine.

### 2.3. Statistical Analysis 

When conducting descriptive statistics, depending on the type of data distribution, for all continuous variables, the mean and its confidence intervals were calculated; in the samples the distribution of which differed from the normal, the median and interquartile range were calculated.

In order to compare the main indicators of the activity of the ambulance service by years, Friedman’s two-way analysis of variance by ranks was used for related samples. The calculation of the Friedman criterion was carried out to compare variables that have a distribution other than normal, according to the Shapiro–Ulik criterion. For group comparison, a systematic comparison of post hoc analysis was applied using post hoc Bonferroni corrections.

In samples, whose distribution was not statistically significantly different from the normal distribution, according to the Shapiro–Ulik test, analysis of variance (ANOVA) for related variables was used. To analyze the dynamics over the years, we used Pillai’s trace criterion. For pairwise comparisons, a post hoc comparison test with Sidak correction was used.

The *p*-value < 0.05 was taken as statistically significant. Statistical analysis of the data was carried out using SPSS version 20.0 (IBM Ireland Product Distribution Limited, Dublin, Ireland).

## 3. Results 

Table 1 shows the structure of ambulance calls: all calls to console 103, canceled calls, consultations, ineffective calls, and fulfilled calls.

Emergency calls are all incoming calls to remote control 103.

Cancelled calls are calls in which the patient’s condition improved and the person canceled the call or decided to go to the hospital on their own without waiting for an ambulance.

An ineffective EMS call is a case of a call regarding a disease, accident, injury, poisoning or other condition that poses a threat to the patient’s life, as a result of which the patient was not there; the call was false (ambulance was not called to this address); the patient was not found the address indicated when calling; the patient turned out to be practically healthy and did not need help; the patient died before the arrival of the EMS team; the patient was taken away before the arrival of the EMS team; the patient was served by a doctor at the clinic before the arrival of the EMS team; or the patient refused to be examined.

Fulfilled calls are ambulance calls as a result of which emergency care was provided to the patient and the patient was transferred for further observation to a local clinic, or the patient was hospitalized in a specialized hospital.

As shown in Table 1, the average number of calls per region per year, as well as the number of calls per 1000 population, did not change significantly from 2019 to 2023.

Call cancellations trended downward from 2724 to 95.5 (2019, 2023), but the data do not show statistical significance. The number of consultations doubled from 2019 to 2023, but the data also do not show statistical significance. The pandemic has taught medical workers how to use healthcare resources economically. Therefore, the number of telephone consultations has increased mostly since 2021. The number of ineffective calls did not change significantly from 2019 to 2023. The number of calls served gradually decreasing from 489,403 to 337,181.5, but the data are also not reliable.

When considering the call structure, all emergency cases were divided into 11 categories (Table 2): infections, cardio-vascular disorders, trauma and poisoning, traffic accidents, obstetrics and gynecology, neurological, respiratory, gastrointestinal, acute surgical, urinary tract diseases, and others. 

According to Table 2, infectious and the urinary system diseases decreased by half from 2019 to 2023, but the data were not statistically significant. Cardiovascular, trauma and poisoning, traffic accidents, neurological, gastrointestinal, obstetric-gynecological, acute surgical pathology, and other diseases did not change significantly. Respiratory diseases decreased from 99,125 to 95,027.6 during the first wave of the CVI (2020), then increased during the second, third, and fourth waves of the CVI to 122,482.28, but these data do not have statistical significance.

When analyzing the structure of calls per region, calls of the first category of urgency (resuscitation is required for life-threatening conditions); calls of the second category of urgency (a patient’s condition that offers a potential threat to life if not treated); calls of the third category of urgency (a patient’s condition that poses a potential threat to health without medical care); and calls of the fourth category of urgency served by emergency medical services teams at primary health care facilities (there is no current or potential threat to the patient’s life and health), no changes were observed. Patients of category IV urgency served by ambulance decreased by 2 and 1.7 times in 2020 and 2021, apparently associated with quarantine measures. There is a significant increase of IV category calls in 2022 and 2023 of 4.7 and 4.5 times, respectively, in comparison with 2019.

In total, 1,498,668 cases of COVID-19 were registered in Kazakhstan over the entire period of the pandemic, of which 19,071 were deaths. A total of 33,563,811 vaccinations and 11,575,012 PCR tests were performed [7].

According to this study, there was a decline in the frequency of category IV EMS calls the COVID-19 pandemic, followed by an increase in frequency during the post-pandemic period.

## 4. Discussion 

The COVID-19 pandemic has dramatically affected emergency ambulance services. According to the literature, the emergency work of the ambulance service during the COVID-19 pandemic showed that there was no significant increase in activity, possibly due to the introduced quarantine measures (for example, due to a decrease in the number of injuries due to vehicle collisions) and also due to the avoidance of medical services [14,15].

When COVID-19 first began to spread in Kazakhstan, the health system recommended adequate measures to control the epidemic. Compliance with quarantine measures has led to a decrease in ambulance calls and morbidity throughout Kazakhstan. In April 2020, there was a sharp decrease in deaths due to the introduction of quarantine, when people were closed up in their homes; the number of deaths due to limited mobility, injuries, and traffic accidents decreased [11].

Kazakhstan used quarantine at an early stage and paired it with other community safety measures, including closing all schools, limiting travel, and disinfecting and sanitizing public areas. Preliminary quarantine measures in Kazakhstan led to social tension, as people could not remain unemployed for a long time [9]. The government was forced to ease quarantine measures, which led to a sharp increase in the incidence of coronavirus. A study conducted in Australia showed that, during quarantine, there was a decrease in the number of calls, but there was a deterioration in response times for ambulances [16]. A study in Polland also illustrated a significant decrease in the number of EMT interventions [17]. Other studies have shown that during the COVID-19 pandemic in 2021, the incidence of difficult-to-transfer cases increased compared to 2019 [18].

This study result was also confirmed by our research in terms of calls of category IV urgency, since during the COVID-19 pandemic there was a decrease in the number of calls. According to the data presented, calls of category IV urgency decreased by 2 and 1.7 times (in 2020 and 2021) during the COVID-19 pandemic, compared to 2019 before the pandemic. The decrease in calls in 2020 during the first wave of coronavirus infection may be related to quarantine measures. The lack of an increase during waves two, three, and four of COVID-19 in 2021, when 1500–7600 new cases per day were noted, may be because the population could not reach an ambulance [11] during the peak of incidence or treated themselves. A limitation of our study is the lack of data for unanswered calls.

The sharp increase in ambulance calls in 2022 and 2023 may be due to the weak performance of the local service, as well as the fact that people with chronic comorbid diseases who did not apply during the pandemic for fear of contracting COVID-19, or “hospital bed shortage”, began to seek medical help. Also, after undergoing coronavirus infection (CVI), many elderly patients with chronic diseases experienced exacerbations, and the level of injuries and infections (ARVI, measles) increased after a long quarantine.

When looking at the call structure, almost all categories did not show any statistically marked changes. One of the important links in prehospital care is the dispatch service. During the pandemic, the importance of dispatch has increased. This is evidenced by the two-fold increase in consultations from 2019 to 2023, but the data did not show statistical significance.

In general, the results of the study show a decrease in the number of category IV calls during the coronavirus infection (2020–2021) and an increase in the number of calls during the post-pandemic period (2022–2023). Further studies are needed for investigation post-pandemic emergency medical services.

### 4.1. Challenges during the COVID-19 Outbreak

Due to the unprecedented spread of COVID-19 around the world since December 2019, the healthcare system in Kazakhstan, like other countries, has faced a number of difficulties in treating this disease and attracting related resources, resulting in a wealth of experience and lessons learned.

#### 4.1.1. Incorrect Forecast

In March 2020, the Ministry of Health of Kazakhstan predicted that if quarantine measures were followed, the number of infected people for a three-month period would not exceed 3500 people [19]. However, on 1 July 2020, there was a sharp increase in the incidence of 18,757 new cases per day [11]. The incorrect forecast led to the unpreparedness of the healthcare system of Kazakhstan. 

#### 4.1.2. Inability to Reach an Ambulance

A sociological survey conducted by Sanj Research Center (2020) showed that 24.5% of respondents in need of emergency assistance faced difficulties in calling an ambulance, 5.5% of calls went unanswered, and 25% of respondents got through but with difficulty. In rural areas, it was tough to reach emergency services [11].

#### 4.1.3. Ambulance Shortage and Shortage of Personal Protective Equipment, Ventilators, Diagnostic Tools (PCR)

Given the acute shortage of ambulances, the Ministry of Health purchased 807 ambulances during the peak of the first wave of coronavirus infections [11]. Medical professionals are on the front line fighting the COVID-19 health crisis. At the beginning of the pandemic, personal protective equipment (PPE) was not purchased promptly, causing a huge number of medical workers to fall ill [9]. Usage of PPE by the community and healthcare providers is crucial for the prevention of the spread of infection and must be available to the public. Kazakhstan’s population and medical institutions faced shortages of medicines and ambulances, ventilators, diagnostic equipment (PCR tests), and beds during the first wave of the pandemic with rising of CVI incidence [9]. Extended hospitalization times caused by bed scarcity have caused delays in the duties of emergency personnel. Disruption in the supply chain of equipment leads to spread of infection and more severe equipment deficiency.

#### 4.1.4. Inadequate Prevention in Society

As crowds are often present during religious and cultural events in Kazakhstan, this has been a determining factor and has made social distancing strategies more challenging. Most people found it impossible to adhere to the quarantine and carried on their business at the local level because of the Kazakhstan government’s inadequate financial support and national protection program. Due to the near-impossibility of maintaining social distance and limiting exposure—two key strategies for lowering the risk of the sickness—it was difficult to control the situation. Violation of quarantine measures was aligned with an increase in the incidence of COVID-19 in the summer of 2020. At the same time, according to studies conducted by Kazakh scientists, 50% compliance with quarantine measures would have led to a decrease in the number of hospitalized up to 9.31 thousand cases during the peak of the pandemic [20].

#### 4.1.5. Shortage of Doctors and Medical Personnel

From 2017 to 2021, the shortage of medical personnel doubled from 10 thousand to 23 thousand units [21]. Due to the shortage of doctors, remaining doctors are forced to work beyond the norm.

#### 4.1.6. Mental State of Medical Personnel

A brief review of the impact of COVID-19 on the reported mental health and well-being of ambulance service personnel revealed higher rates of distress, PTSD, and insomnia [22]. A national survey in the United Kingdom of ambulance service clinicians and paramedics preparedness and response to the COVID-19 pandemic also showed the psychological distress of most participants [23].

The Center for Evidence-Based Medicine provides data on mortality per 100 thousand people in the different countries. It was demonstrated that excess mortality in Kazakhstan was very high in 2020 in comparison with developed countries; it reached 21% compared to the previous three years [24]. The COVID-19 pandemic has significantly affected the health status of the population of Kazakhstan, as well as the well-established process of providing medical care in the country, and the unpreparedness of the healthcare system’s response to emergencies has been revealed [24]. Regrettably, Kazakhstan’s COVID-19 pandemic, like those in other nations, has highlighted the shortcomings of the country’s healthcare system, including its lack of public healthcare, delayed reaction time, disregard for the needs of the population’s most vulnerable members, and weak resistance to disinformation [11].

## 5. Conclusions

The results of the study do not contain changes in the dynamics of ambulance calls, except for calls of category IV urgency. A significant decrease of category IV urgency calls were observed during the pandemic of COVID-19 infection. The increase in the category IV calls in 2022 and 2023 that did not require emergency assistance may be due to the poor performance of local services, and an increase in exacerbations of chronic diseases as well as infections.

The lesson we must learn from this pandemic is the need to be carefully prepared as the worst-case scenario unfolds. A national pandemic preparedness plan must be developed in Kazakhstan in order to get ready for the next outbreak.

1. Finances: The ability to raise the money required in the event of a new epidemic is crucial to maintain services.
2. During a pandemic, all parts of the healthcare system must work harmoniously. The weak performance of one link leads to the overload of another. For ambulances to operate effectively, it is necessary to improve the work of local outpatient services.
3. Coordinate the proper distribution of resources. Investing in trained doctors and paramedics, developing proprietary medications, vaccines, and medical supplies. Training and developing the practical skills of medical personnel when working with especially dangerous infections (personal protective equipment, sorting patients by severity, algorithms for diagnosing and providing emergency care, transportation). Expanding our own manufacturing of masks and personal protective equipment. Improving the equipment of ambulances with ventilators, oxygen concentrates, and medicines.
4. The lack of preparedness of the ambulance service in Kazakhstan is associated with an incorrect forecast. It is necessary to use prognostic scales to correctly predict morbidity.
5. The experience of the pandemic has shown the high cost-effectiveness and safety of distant consultations. It is necessary to further develop dispatch system, telemedicine, and distant consultations.

## Figures and Tables

**Table 1:** Numbers of emergency calls (2019–2023).

Number of Emergency Calls	2019 †	2020 †	2021 †	2022 †	2023 †	*p*
Emergency calls (mean for one region per 1 year)	529,307 (±381,301)	450,940 (±334,591)	494,578 (±350,816)	380,680.5 (±366,475)	365,157 (±366,521)	0.726
Emergency calls per 1000 inhabitants *	466.58 (±137.81)	426.10 (±126.77)	470.55 (±127.36)	436.69 (±105.83)	410.02 (±104.85)	0.021
Cancelled calls (mean for one station per 1 year)	2724 (±8231)	1987 (±8700)	1112.5 (±5765)	357.5 (±5622)	95.5 (±2954)	0.647
Number of consultations (mean for one station per 1 year)	7635 (±15,516)	8516 (±15,361)	13,616 (±13,391)	14,069 (±26,221)	16649 (±40,014)	0.657
Ineffective calls (mean for one regio per 1 year)	8905 (±8644)	9985 (±6801)	12,525 (±8460)	9789 (±9193)	10,853.5 (±11,327)	0.829
Fulfilled calls (mean for one station per 1 year)	489,403 (±304,808)	416,183 (±281,659)	455,750 (±324,041)	335,520 (±318,337)	337,181.5 (±324,698)	0.79

* Me (IQR). † Plus–minus values are means ± SD. IQR denotes interquartile range.

**Table 2:** Causes and categories of emergency calls (2019–2023).

Clinical Diagnosis	Monthly Statistics					*p*
	2019 †	2020 †	2021 †	2022 †	2023 †	
Infectious Diseases	26,365 (±39,327)	18,678 (±32,957)	15,903 (±24,630)	15,373 (±19,918)	12,520 (±15,408)	0.153
Cardio-vascular diseases	72,543 (±66,100)	72,711 (±68,094)	70,879.38 (±52,833)	65,472.5 (±52,468)	69,820.5 (±46,398)	0.938
Trauma and poisoning	27,474 (±34,891)	27,315 (±34,021)	29,702 (±33,141)	27,093 (±30,811)	27,716.5 (±30,423)	0.97
Traffic accidents	1518 (±2152)	1432 (±1465)	1659 (±1930)	1527.5 (±1788)	1633.5 (±1252)	0.676
Neurological diseases *	37,277.24 [26,906.68; 47,647.79]	30,826.5 [22,784.17; 38,868.83]	34,613.88 [25,581.67; 43,646.08]	36,726.44 [26,627.31; 46,825.58]	37,083.9 [26,256.08; 47,911.72]	0.899
Respiratory diseases *	99,125 [71,767.09; 126,482.91]	95,027.58 [70,391.22; 119,663.93]	122,482.28 [90,711.49; 154,253.07]	123,817.72 [87,691.84; 159,943.6]	104,206.15 [77,571.01; 130,841.29]	0.677
Gastrointestinal diseases	23,872 (±18,754)	19,900 (±15,152)	23,196 (±23,105)	23,554 (±26,680)	27,854.5 (±24,857)	0.719
Obstetrics and gynecology	20,924 (±35,571)	21,935 (±37,090)	23,890 (±35,826)	20,382.5 (±22,379)	17,848.5 (±24,134)	0.911
Acute surgical diseases *	17,182.59 [13,294.11; 21,071.06]	12,234.5 [9441.24; 15,027.76]	13,252.65 [9687.71; 16,817.58]	13,460.5 [9627.96; 17,293.04]	11,592.9 [8492.48; 14,693.32]	0.183
Urinary tract diseases	11,399 (±14,691)	8083 (±12,309)	7053 (±7277)	6837.5 (±7999)	6290.5 (±7518)	0.046
Others	80,463 (±67,543)	67,060 (±65,008)	76,365 (±50,626)	75,979.5 (±52,343)	65,662.5 (±55,830)	0.622
Category of urgency						
I urgency category	15,885 (±34,957)	15,857 (±23,809)	18,754 (±20,831)	14,633.5 (±20,790)	13,604.5 (±20,385)	0.901
II urgency category	119,466 (±134,124)	98,817 (±118,520)	116,745 (±112,724)	109,072.5 (±94,825)	105,274.5 (±81,443)	0.943
III urgency category	129,696 (±87,978)	111,778 (±75,081)	112,674 (±108,103)	110,524 (±116,393)	96,230 (±105,434)	0.702
IV urgency category, served by ambulance (abs number)	36,153 (±86,113)	17,583 (±44,670)	20,375 (±52,019)	172,420.5 (±147,203)	161,536 (±121,573)	0.001
IV urgency category, served by emergency medical services teams at primary health care facilities (abs. number)	91,207 (±103,525)	108,512 (±121,601)	117,501 (±138,812)	120,174.5 (±137,539)	94,864 (±123,835)	0.629

* Me (IQR). † Plus–minus values are means ± SD. IQR denotes interquartile range.

## Data Availability

The datasets generated for this study are available on request to the corresponding author.
